# Risk of breast cancer in Lynch syndrome: a systematic review

**DOI:** 10.1186/bcr3405

**Published:** 2013-03-19

**Authors:** Aung Ko Win, Noralane M Lindor, Mark A Jenkins

**Affiliations:** 1 Centre for Molecular, Environmental, Genetic and Analytic Epidemiology, Melbourne School of Population and Global Health, The University of Melbourne, 207 Bouverie Street, Parkville, VIC 3010 Australia; 2 Department of Health Science Research, Mayo Clinic Arizona, 13400 E. Shea Blvd., Scottsdale, AZ 85259 USA

## Abstract

**Introduction:**

Lynch syndrome is an autosomal dominantly inherited disorder of cancer susceptibility caused by germline mutations in the DNA mismatch repair (MMR) genes. Mutation carriers have a substantial burden of increased risks of cancers of the colon, rectum, endometrium and several other organs which generally occur at younger ages than for the general population. The issue of whether breast cancer risk is increased for MMR gene mutation carriers has been debated with evidence for and against this association.

**Methods:**

Using the PUBMED, we identified all relevant studies of breast cancer associated with Lynch syndrome that were published by 15 December 2012. In the review, we included: (i) molecular studies that reported microsatellite instability and/or immunohistochemistry in breast cancer tumors of MMR gene mutation carriers; and (ii) risk studies that investigated risk of breast cancer for confirmed MMR gene mutation carriers or families or clinically and/or pathologically defined Lynch syndrome families.

**Results:**

We identified 15 molecular studies and, when combined, observed 62 of 122 (51%; 95% CI 42 to 60%) breast cancers in MMR gene mutation carriers were MMR-deficient. Of the 21 risk studies identified, 13 did not observe statistical evidence for an association of breast cancer risk with Lynch syndrome while 8 studies found an increased risk of breast cancer ranging from 2- to 18-fold compared with the general population (or non-carriers). There is only one prospective study demonstrating an elevated risk of breast cancer for MMR gene mutation carriers compared with the general population (standardized incidence ratio 3.95; 95% CI 1.59, 8.13).

**Conclusions:**

Since breast cancer is a relatively common disease in the general population, more precise estimates of risk and gene-specific risks will need to utilize large prospective cohort studies with a long follow-up. While current data are inconclusive at a population level, individual tumor testing results suggest that MMR deficiency is involved with breast cancers in some individuals with Lynch syndrome.

## Introduction

Lynch syndrome, formerly known as hereditary nonpolyposis colorectal cancer (HNPCC) [1], is an autosomal dominantly inherited disorder of cancer susceptibility caused by germline mutations in the DNA mismatch repair (MMR) genes, *MLH1, MSH2, MSH6 *and *PMS2*. The prevalence of individuals who carry a pathogenic germline mutation in one of these genes in the population is estimated to be, depending on various assumptions, from 1 in 370 to 1 in 3,000 [2-4]. Though rare, mutation carriers have a substantial burden of increased risks of cancers of the colon, rectum, endometrium, stomach, ovary, ureter, renal pelvis, brain, small bowel, and hepatobiliary tract which generally occur at younger ages than for the general population [5].

Lynch syndrome-associated cancers typically exhibit DNA microsatellite instability (MSI) and loss of MMR protein expression. Instability of microsatellite DNA sequences characterized by the presence of random contractions or expansions in the length of simple sequence repeats, can be detected by polymerase chain reaction-based methods [6,7]. The loss of MMR protein expressions can be detected by immunohistochemistry (IHC) [5]. These techniques have been widely used to triage colorectal cancer patients for testing for germline mutations in the MMR genes, and recently cancers at other sites [8]. As a consequence, various MMR-deficient tumors have been identified in organs that are not traditionally recognized as part of the spectrum of Lynch syndrome.

These cancers include adrenocortical adenocarcinoma [9-11], thyroid carcinoma [10], peritoneal mesothelioma [11], malignant fibrohistiocytoma [12,13], rhabdomyosarcoma [14], dermatofibrosarcoma [15], leiomyosarcoma [16-19], liposarcoma [19], carcinoid tumour [20], non-Hodgkin lymphoma [21], malignant melanoma [22], pancreas [11,23], prostate [24] and breast cancers [25-39]. However, for these tumors except pancreatic cancer [40], an increase in risk for MMR gene mutation carriers compared with the population (or non-carriers) has not been observed.

Initially raised by Henry Lynch and colleagues several decades ago [41], the issue of breast cancer risk in Lynch syndrome has been debated with studies providing evidence for and against breast cancer being part of Lynch syndrome [42-44]. In this article, we have reviewed the existing studies that investigated whether breast cancer is caused by a mutation in an MMR gene or whether the risk of breast cancer is increased for women who carry a mutation in an MMR gene.

## Materials and methods

### Identification of studies

We used PUBMED [45] to search for all relevant studies of breast cancer associated with Lynch syndrome that were published by 15 December 2012. The following combinations of key words were used: 'breast cancer/carcinoma/tumor or extracolonic cancer/tumor/neoplasia' and 'mismatch repair gene mutation or Lynch syndrome or HNPCC'. No language restrictions were imposed. References from relevant articles, letters, reviews and editorials were searched to identify any additional relevant studies. Studies were reviewed initially on the basis of title and abstracts, and then all full manuscripts for those that appeared relevant were obtained and checked for eligibility.

### Eligibility criteria

For molecular studies, studies were eligible if they reported mismatch repair deficiency status (either by MSI and/or IHC) of breast cancer tumors of MMR gene mutation carriers. For risk studies, studies were eligible (i) if they were case-control or cohort studies, and (ii) if they investigated the risk of breast cancer for confirmed MMR gene mutation carriers or families or clinically and/or pathologically defined Lynch syndrome families compared with non-carriers or the general population. Studies were excluded if they were editorials or literature reviews.

### Data extraction

For molecular studies, we extracted the following data: first author's name, year of publication, country in which the study was performed, number of breast cancers observed in MMR gene mutation carriers, specific mutated MMR gene, age at diagnosis of breast cancer, and the MSI and/or IHC status of the breast tumor.

For risk studies, we extracted the following data: first author's name, year of publication, country in which the study was performed, recruitment and ascertainment method of families or mutation carriers, definition of Lynch syndrome, numbers of MMR gene mutation carriers, specific mutated MMR gene, observed and expected numbers of breast cancers, estimates of breast cancer risk for mutation carriers compared with non-carriers or the general population (expressed as standardized incidence ratio, odds ratio, relative risk or hazard ratio) and absolute or cumulative risk of breast cancer for mutation carriers (expressed as lifetime risk or 10-year risk).

### Statistical analysis

For molecular studies, we defined a tumor with high level of microsatellite instability (≥ 30% of markers tested) or those with loss of normal expression of any of the four DNA MMR genes by IHC to be a MMR-deficient tumor. We divided the total number of MMR-deficient breast cancers by the total number of breast cancers in MMR gene mutation carriers to estimate the proportion of breast cancers in MMR gene mutation carriers that were MMR-deficient.

For risk studies that did not provide the relative risk (or standardized incidence ratio), we calculated the estimated the standardized incidence ratio (SIR) by dividing the observed numbers of breast cancers by the reported expected numbers. When not provided by the study, we calculated the 95% CI assuming that the numbers of observed cases followed a Poisson distribution. All statistical analyses were conducted using Stata 11.2 [46].

## Results

**Figure 1:**
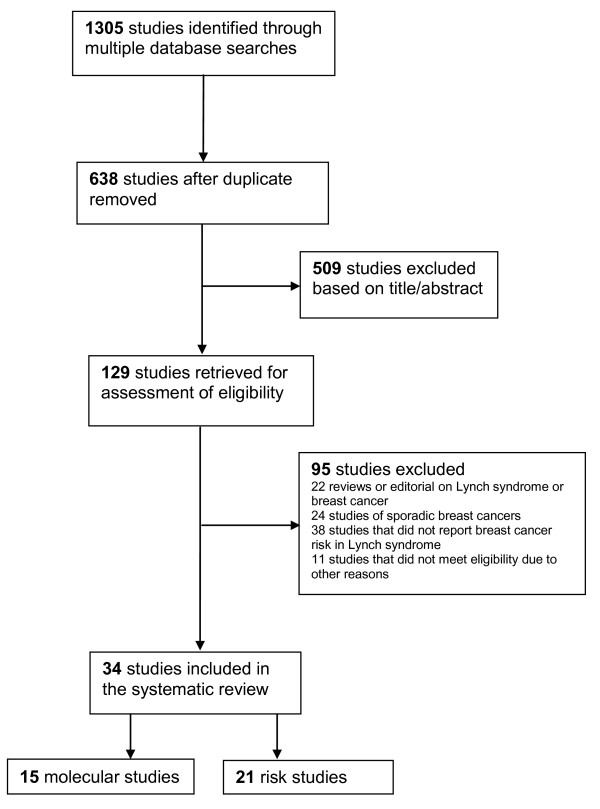
**Flow diagram of the selection of studies**.

We identified 638 studies identified through the literature search, and retrieved 129 of these studies for full assessment for eligibility. A total of thirty-four studies were included in the systematic review including fifteen molecular studies and twenty-one risk studies (two studies were both molecular and risk studies) (Figure 1).

**Figure 2:**
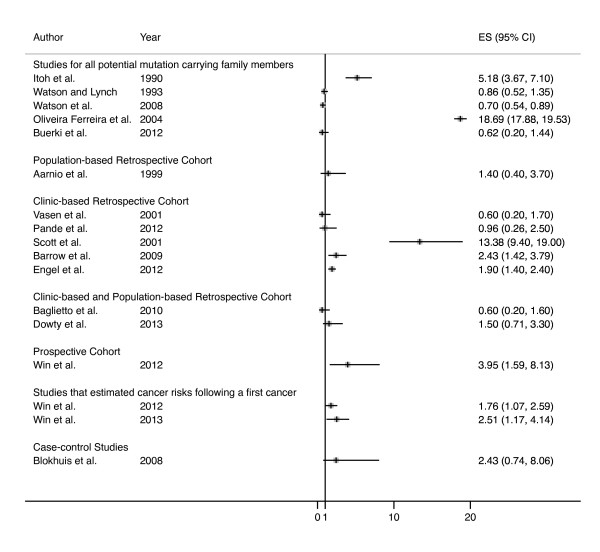
**Estimates of risk of breast cancer in Lynch syndrome compared with the general population (or non-carriers of mismatch repair gene mutation)**. ES, effect size.

Of the fifteen molecular studies, five were single case reports [25-29] and ten were case series [30-39] (Table 1). When all case series were combined, 62 of 122 (51%, 95% CI 42%, 60%) breast cancers in mutation carriers were MMR-deficient. Of the twenty-one risk studies [33,37,47-65], thirteen did not show elevated risks [33,37,48-50,52-57,61,62], while eight did observe an increased risk of breast cancer in Lynch syndrome [47,51,58-60,63-65] (Additional file 1). Eight of the risk studies estimated risk retrospectively for all known and potential mutation carrying family members combined [37,47-53], nine studies estimated risk for mutation carriers retrospectively [54-62], one study estimated risk for carriers prospectively [63], two studies estimated cancer risk for carriers who had a previous cancer diagnosis [64,65], and one study compared risk of breast cancer in known carriers to the risk in their known mutation-negative (non-carrying) sisters [33]. Three of the risk studies did not quantify the risk of breast cancer [50,52,56] and one study only compared the proportion of tumors that were breast cancer in carriers compared with the population [53]. For five studies, we calculated the SIR based on the ratio of the reported observed and expected numbers of cases [33,37,49,51,59] and for one additional study we estimated the 95% CI for the association [48]. The estimated risks of breast cancer in Lynch syndrome compared with the general population (or non-carrier) are shown in Figure 2, and the strength of association ranged approximately between 2- and 18-fold.

**Table 1:** Summary of molecular studies that investigated mismatch repair (MMR) deficiency status of breast cancer in Lynch syndrome

Single case reports					
Author	Year	Country	Method	MMR gene mutated/absence of MMR protein in IHC/MSI status	Age, years

Boyd *et al. *[25]	1999	USA	MSI, IHC	*MLH1*/MLH1 loss/MSI-H	71 (male)
Caluseriu *et al. *[26]	2001	Italy	MSI	*MLH1*/NR/MSS	
Westenend *et al. *[27]	2005	The Netherlands	MSI, IHC	*MSH2*/MSH2 and MSH6/MSI-H	49
Akoum *et al. *[28]	2009	Lebanon	MSI, IHC	*MSH2*/MSH2 and MSH6/MSS	26
D'Arcy *et al. *[29]	2011	USA	IHC	*MSH2*/MSH2 and MSH6/NR	74

**Case series**					

Author	Year	Country	Method	Number of MMR-deficient breast cancers/ number of MMR gene mutation carriers	Age, years, mean ± SD

Risinger *et al. *[30]	1996	USA	MSI, IHC	3/5 (60%)	NR
Muller *et al. *[31]	2002	USA	MSI, IHC	0/3 (0%)	NR
de Leeuw *et al. *[32]	2003	The Netherlands	MSI	7/11 (64%)	43.6 ± 9.2
Blokhuis *et al. *[33]	2008	South Africa	MSI, IHC	5/6 (83%)	51.8 ± 16.9
Shanley *et al. *[34]	2009	Australia	MSI, IHC	3/4 (75%)	65.0 ± 12.3
Jensen *et al. *[35]	2010	Denmark	IHC	7/16 (44%)	50.4 ± 13.1
Walsh *et al. *[36]	2010	Australia, USA, Canada	MSI, IHC	18/35 (51%)	57.5 ± 8.1
Buerki *et al. *[37]	2012	Switzerland	MSI, IHC	6/7 (86%)	53.7 ± 15.2
Lotsari *et al. *[38]	2012	Finland	MSI, IHC	13/20 (65%)	52.7 ± 7.0
Grandval *et al. *[39]	2012	France	MSI	0/15 (0%)	53.1 ± 10.0

IHC, immunohistochemistry; MSI, microsatellite instability; MMR, mismatch repair; MSI-H, high microsatellite instability; MSS, microsatellite stable; NR, not reported.

## Discussion

We identified 34 published studies that have reported data that can be used to address the question of whether breast cancer is a Lynch syndrome cancer. In this review, we have attempted to combine all accumulated published evidence to answer the question. There appears to be some evidence from the epidemiological risk studies favoring the concept of an increased risk of breast cancer for germline MMR gene mutation carriers, though the mechanism cannot yet be known.

### Molecular studies

Overall, the 15 studies found that approximately half of the breast cancer tumors in carriers of MMR gene mutations were MMR-deficient. Further, the largest case series demonstrated that the proteins not expressed corresponded to the gene that was mutated in the carrier [36]. They also observed that the MMR-deficient breast cancers had pathology features that were consistent with the features identified in colorectal cancers caused by MMR gene mutations [66,67], that is, MMR-deficient breast cancers were more likely to be poorly differentiated, have a higher mitotic rate, peritumoral lymphocytes, confluent necrosis, a solid growth pattern, and more likely to be estrogen receptor-negative and progesterone receptor-negative than the MMR-proficient breast cancers.

These data support the hypothesis that the MMR gene mutation carriers may have been predisposed to develop these particular breast cancers; that is, MMR gene mutations may initiate the development of breast cancer (as a driver) or accelerate carcinogenesis in breast cancers that occur in MMR gene mutation carriers (as an accelerator). However, these observations are also consistent with MMR deficiency being a phenotype arising within breast cancer that was caused by another factor (as a passenger), rather than the phenotype driving or accelerating carcinogenesis.

### Risk studies

One method to overcome this problem of cause or consequence is to determine whether breast cancer is more likely to develop in carriers of MMR gene mutations compared with non-carriers (or the general population), that is, empirical evidence of risk. Eight studies [37,47-53] estimated breast cancer risk for all female relatives of MMR gene mutation carriers not known to be a non-carrier, that is, those identified as carriers as well as those who were not tested for genetic mutations. Therefore, their study estimates are for an unknown distribution of carriers and non-carriers. If there is an increased risk of breast cancer for MMR gene mutation carriers, their estimates will therefore be attenuated, that is, they will underestimate the true risk of breast cancer for carriers [60]. This may be a reason that all of these studies [37,48-50,52,53] except two [47,51] observed no evidence of an increased risk of breast cancer or observed a reduced risk of breast cancer for Lynch syndrome families.

One retrospective study [54] compared the observed number of breast cancer in confirmed and obligate carriers with the number expected, and they observed no evidence of an increased risk of breast cancer for carriers (SIR 1.4, 95% CI 0.4, 3.7). As they ascertained carriers from a population cancer registry, the study is less likely to have ascertainment bias because of family history. The lack of evidence in the study might be due to the small sample size (360 mutation carriers).

Six studies [55-60] estimated risk of breast cancer for the female relatives of families ascertained through genetics clinics. Of these, three [55-57] observed no evidence of an increased risk of breast cancer while three [58-60] observed an approximately 2- to 13-fold increased risk of breast cancer for MMR gene mutation carriers compared with the general population. Persons attending genetics clinics are often referred because of a strong family history of cancer. Because these studies did not attempt to correct for the family history ascertainment in their statistical analyses, the estimates of breast cancer risk are likely to be upwardly biased [68] if any of the family members attended the clinics because of a family history of breast cancer.

Two studies [61,62] from the Colon Cancer Family Registry estimated risk of breast cancer for MMR gene mutation carriers and found no evidence of an increased risk. The study samples were families ascertained through genetics clinics as well as population cancer registries. In these studies, the analyses were adjusted for how these families were ascertained. As it is very difficult to know the exact reason(s) influencing a person/family to be referred and/or attend a genetics clinic, the correction for ascertainment is usually conservative, that is, by conditioning on the cancer status of all family members to avoid overestimation of any particular cancer risk that may have, in part, contributed to referral or attendance [68]. The consequence of this method is that, if families are ascertained only because of colorectal and/or other cancers but not breast cancer, the power to identify an increased risk for breast cancer will be reduced and breast cancer risk estimates from these studies are likely to be underestimated.

Prospective studies avoid the ascertainment issue described above because the carriers used to estimate cancer risk are unaffected at the time of recruitment or first observation. To date, there is only one prospective study of MMR gene mutation carriers without prior diagnosis of cancer [63]. An increased risk of breast cancer was observed In that study for MMR gene mutation carriers (SIR 3.95, 95% CI 1.59, 8.13). An alternative explanation, for at least part of the observed increase in breast cancer risk in this prospective cohort study, is that it might be due to screen-detected cases. Mammographic screening may be more prevalent and/or frequent in women from Lynch syndrome families who are more concerned about cancer risk compared with the general population, thus resulting in breast cancer diagnoses being brought forward due to early detection. If this hypothesis was true, it might be expected to be more evident in prospective studies, given that observation time for risk used in these analyses begins from when the family was first seen in a clinic, the same time during which an increased awareness of cancer risk may arise.

The two other studies of the Colon Cancer Family Registry observed an approximately 2-fold increased risk of breast cancer for MMR gene mutation carrying women. These included those who had a previous diagnosis of colorectal cancer [64] or endometrial cancer [65]. Such studies may be more powerful to identify an increased risk if those with a previous cancer diagnosis represent a more cancer-susceptible subset of carriers.

Blokhius *et al. *[33] compared the number of breast cancer diagnoses observed in 87 carrier women (cases) with that for their 121 non-carrier sisters (controls) of *MLH1 *c.C1528T mutation. In this study design, unaffected siblings of familial cases have the same familial risk profile (that is, they could share all other multifactorial causes with their gene-carrying siblings) and therefore, share potential confounders that are correlated within siblings, in other words, it is an informative comparison within the same study base [69]. The lack of evidence in the study might be due to the small sample size given that at least 700 carriers and 700 non-carriers are needed to have 90% power to detect an odds ratio of 2 or greater at the 0.05 level of significance, assuming the 5% prevalence of breast cancer in non-carriers.

### Is breast cancer a Lynch syndrome cancer?

The estimates of an increased risk of breast cancer in Lynch syndrome described in the previous studies are relatively small compared with the increased risks observed for colorectal and endometrial cancers [70]. However, even a small increase in breast cancer risk might point to interesting biological connections. Vasen *et al. *[55] hypothesized, '...because of the defect in the MMR system, mutations may accumulate in genes involved in the progression of breast cancer. The accumulation of mutations may lead to acceleration of tumor development...'. The later hypothesis is supported by Walsh *et al. *[71] who observed that microsatellite unstable breast ductal carcinoma *in situ *were predominantly of higher nuclear grade, suggesting that aberrations in MMR function may lead to a more aggressive phenotype in breast cancer.

The suggested small increased risk of breast cancer in Lynch syndrome is important to confirm, given that breast cancer is a common disease in women. The average lifetime risk of breast cancer in women is estimated to be 12.2% [72]. The threshold risk of breast cancer for recommended magnetic resonance imaging (MRI) screening of the breast suggested by the American Cancer Society is a lifetime risk of 20-25% or greater [73]. If the 2-fold increased risk of breast cancer in Lynch syndrome compared with the general population (estimated by previous studies [63-65]) is confirmed, then consideration of screening with breast MRI would be indicated for women carrying MMR gene mutations. Further, even if MMR deficiency does not prove that MMR mutation causes breast cancer, the 51% prevalence of the phenotype in breast cancers in carriers would strongly support the clinical feasibility of testing breast cancer tumors in families suspected of segregating an MMR gene mutation, where no colorectal tumors are available for testing. In this circumstance, a breast tumor showing loss of expression of an MMR gene by IHC would indicate a significant likelihood of an underlying germline mutation, while a normal MMR gene result would not rule out Lynch syndrome.

Most of the previous studies estimated risk of breast cancer for all MMR gene mutation carriers combined. Some studies [37,53,55,58,59,62,64,65] attempted to test for differences in breast cancer risk by MMR genes; for example, Scott *et al. *[58] observed an increased risk of breast cancer for *MLH1 *mutation-carrying families but not in *MSH2 *families (see Additional file 1 for details). Although there is indeed some suggestion that breast cancer risk may vary by gene, there have been no formal attempts by the previous studies to examine gene- and age-specific risks for breast cancer. This may be an important consideration for future, adequately powered studies, given the heterogeneity of risk by gene that exists for other cancers in Lynch syndrome [61,70,74-76]. Currently there has been insufficient power to identify differences, as indicated by the overlapping confidence intervals between gene-specific cancer risks. Until these data are available and the effectiveness of screening tools for carriers is known, it is not possible to suggest the optimal screening strategies for breast cancer in Lynch syndrome.

## Conclusions

It is premature to provide a definitive answer to whether breast cancer is a Lynch syndrome cancer because of the following limitations. There is only one prospective study demonstrating an elevated risk of breast cancer in Lynch syndrome and further independent evidence is required to confirm the findings. Given that previous epidemiological studies used different selection methods, subjects and statistical methods, a meta-analysis is not appropriate to generate a pooled estimate for breast cancer risk. Current evidence of breast cancer risk is mainly from Caucasian populations and therefore may not be applicable to other ethnicities or populations, particularly given that previous studies have demonstrated that risk of Lynch syndrome cancers vary across populations [77]. Given these limitations, and given that breast cancer is a relatively common disease in the general population, we recommend that studies to provide more precise estimates of risk will need to utilize large prospective cohort studies with a long follow-up. While current data are inconclusive at a population level, individual tumor testing results suggest that MMR deficiency is involved with breast cancers in some individuals with Lynch syndrome.

## Abbreviations

IHC: immunohistochemistry; HNPCC: hereditary nonpolyposis colorectal cancer; MMR: mismatch repair; MRI: magnetic resonance imaging; MSI: microsatellite instability; SIR: standardized incidence ratio.

## Competing interests

The authors have no conflict of interest to declare with respect to this manuscript.

## Authors' contributions

AKW carried out study concept and design, literature review, collection and assembly of data, analysis and interpretation of data, drafting and gave approval of the final version of the manuscript for publication. NML and MAJ carried out study concept and design, interpretation of data, drafting and gave approval of the final version of the manuscript for publication.

## Supplementary Material

Additional file 1 **Summary of risk studies that investigated risk of breast cancer in Lynch syndrome**. Brief descriptions of study population, study design, study's major finding(s) for the risk studies included in the review [33,47-65,78-80]. Click here for file
