# Supplementary material for: Risk of breast cancer in Lynch syndrome: a systematic review
Source: Breast Cancer Res. 2013 Mar 19;15(2):R27. doi: 10.1186/bcr3405 (PMC3672741; doi:10.1186/bcr3405)
Supplement: Additional file 1 — Summary of risk studies that investigated risk of breast cancer in Lynch syndrome. Brief descriptions of study population, study design, study's major finding(s) for the risk studies included in the review [33,47-65,78-80]. [file bcr3405-S1.DOC]

Additional File 1. Summary of risk studies that investigated risk of breast cancer in Lynch syndrome.

| Author | Year | Country | Population | Study design | Major finding(s) for breast cancer | Comments |
| --- | --- | --- | --- | --- | --- | --- |
| Itoh et al. | 1990 | UK | 885 first-degree relatives from 130 families of Lynch type II cancer family syndrome.  Ascertained from the families attending St. Mark’s Hospital Family Cancer Clinics because of a strong family history of colorectal cancer and families attending the Royal Free Hospital Genetic Clinic because of a strong family history of breast cancer. | Retrospective cohort study | Observed death from breast cancer=38  E=not reported  SIR^¶^=5.18 (95% CI 3.67-7.1)  Lifetime risk=1 in 3.7 | Study subjects were ungenotyped.  The estimates may not reflect only mutation carriers.  SIR estimates reflect the risk of death from breast cancer for relatives of Lynch syndrome families; NOT the risk (incidence) of breast cancer. |
| Watson and Lynch | 1993 | USA | 1,317 members from 23 high-risk families [at least three first-degree relatives were affected by colon or endometrial cancer; at least two with colon cancer at age ≤50 years]  Families were ascertained because of a strong family history of colorectal cancer. | Retrospective cohort study | O=19  E=22.0  SIR=0.9  Median age at diagnosis=51 years | Study subjects were ungenotyped.  The estimates may not reflect only mutation carriers. |
| Watson et al. | 2008 | Denmark  Holland  Finland  USA | 6,041members (2,926 women) from 261 families with *MLH1* or *MSH2* mutations (1,756 confirmed mutation carriers and ungenotyped first-degree relatives).  All confirmed mutation non-carriers and descendants of confirmed non-carriers were excluded. | Retrospective cohort study | O=65  E= 92.65  SIR=not reported | The estimates may not reflect only mutation carriers. |
| Aarnio et al. | 1995 | Finland | 293 putative* gene carriers from 40 families fulfilled the Amsterdam Criteria I (23 *MLH1* and 1 *MSH2*, and others unknown).  Ascertained through the Finnish HNPCC Registry. | Retrospective cohort study | O=9 (5 first tumors and 4 metachronous tumors)  E=not reported  SIR=not reported  Authors stated “We cannot consider… as an integral part of the HNPCC tumor spectrum…”. | The estimates may not reflect only mutation carriers. |
| Oliveira Ferreira et al. | 2004 | Brazil | 1241 members (652 women) from 29 families (25 Amsterdam Criteria I and 4 Amsterdam Criteria II ).  Ascertained through the Hereditary Colorectal  Cancer Registry of the Pelvic Surgery Department of  the Research and Treatment Center Hospital of Cancer  A.C. Camargo (São Paulo, Brazil). | Retrospective cohort study | O=13 of 49 extracolonic tumors in women (26.5%)  Observed prevalence = 1993.86/100,000  Expected prevalence = 106.66/100,000 (São Paulo, 2002)  SIR=not reported | The estimates may not reflect only mutation carriers. |
| Goecke et al. | 2006 | Germany | 988 members (418 confirmed mutation carriers, 22 obligate** mutation carriers, and 548 first- or second-degree relatives) from 281 families with *MLH1* (n=124) or *MSH2* (n=157) mutations.  Families fulfilled the Amsterdam Criteria II or the original Bethesda guidelines were ascertained through the six German centers. | Retrospective cohort study | O=1% of 600 tumors in *MLH1* families and 0.9% of 781 tumors in *MSH2* families.  E=not reported  SIR=not reported  Authors stated “…no significant genotype-phenotype correlations could be observed”. | The estimates may not reflect only mutation carriers. |
| Geary et al. | 2008 | UK | 723 members from 130 families with *MLH1* (n=62) or *MSH2* (n=64) or *MSH6* (n=4) mutations.  Ascertained through six cancer genetics units in the London region. | Retrospective cohort study | O=37 (8 confirmed, 10 obligate^┼^, 4 phenotypic^┼┼^ carriers and 15 relatives unknown for mutation status)  OR^=1.7 for overall  OR^=1.7 for *MLH1*  OR^=1.8 for *MSH2*  Median age at diagnosis = 50 years (range 33-74) | The estimates may not reflect only mutation carriers. |
| Buerki et al. | 2012 | Switzerland | 632 female members (92 carriers, 61 non-carriers, and others unknown) from 70 families with *MLH1* (n=39) or *MSH2* (n=31) mutations.  Ascertained through patients referred from hospital services and private practices for genetic testing. | Retrospective cohort study | O=13 (2 *MLH1*, 2 *MSH2*, and 9 unknown for carrier status)  E=not reported  SIR=not reported  Cumulative risk to age 70  Overall=5.2% (95% CI 2.2-8.3)  MLH1=2.7% (95% CI 0-5.4%)  MSH2=10.1% (95% CI 2.9-17.4)  Population risk=8.1% | The estimates may not reflect only mutation carriers. |
| Aarnio et al. | 1999 | Finland | 360 mutation carriers (265 confirmed and 95 obligate^#^ carriers) from 50 families with *MLH1* (n=47) or *MSH2* (n=3) mutations.  Ascertained through the population-based nationwide Finnish Cancer Registry. | Retrospective cohort study | O=4  E=not reported  SIR=1.4 (95% CI 0.4-3.7) | No ascertainment bias. |
| Vasen et al. | 2001 | Netherlands | Confirmed or putative^##^ mutation carriers (187 *MLH1*, 141 *MSH2*) from 138 families fulfilled the Amsterdam Criteria or with *MLH1* (n=34) or *MSH2* (n=40) or *MSH6* (n=5) mutations.  Ascertained through the Dutch HNPCC Registry. | Retrospective cohort study | *MLH1* carriers  O=4  E=not reported  SIR=0.6 (95% CI 0.2-1.5)  *MSH2* carriers  O=3  E=not reported  SIR=0.6 (95% CI 0.2-1.7)  Mean age at diagnosis=46 years (range 32–59) | Estimate of association may be upwardly biased.^¶¶^ |
| Parc et al. | 2003 | France | 348 mutation carriers (163 confirmed carrier probands, 153 confirmed carrier relatives, 32 obligate^^ carrier relatives) from 163 families with *MLH1* or *MSH2* mutation carriers.  Ascertained through probands referred to the family cancer clinic and met  the Amsterdam Criteria II . | Retrospective cohort study | O=not reported  E=not reported  SIR=not reported  Authors stated “Of interest was the fact that breast, thyroid,  lung, and prostate cancer were infrequent for both groups of  patients suggesting that patients with a deleterious mutation  in *MSH2* and *MLH1* may not be at increased risk for these cancers.” | Estimate of association may be upwardly biased. ^¶¶^ |
| Pande et al. | 2012 | USA | 368 carriers (152 *MLH1*, 197 *MSH2*, 16 *MSH6* and 3 *PMS2*) (217 women) from 176 families.  Ascertained families from the gastroenterology and gynecologic oncology clinics at MD Anderson Cancer Center and through the genetic counselors at the Clinical Cancer Genetics Clinic. | Retrospective cohort study | All MMR carriers (n=217)  O=5  E=6.94  SIR=0.72 (95% CI 0.23-1.7)  MMR carrier relatives (n=123) i.e. excluded probands  O=4  E=4.17  SIR=0.96 (95% CI 0.26-2.5) | Estimate of association may be upwardly biased. ^¶¶^ |
| Scott et al. | 2001 | Australia | 95 families fulfilled the Bethesda Criteria (n=63) or the Amsterdam Criteria (n=32).  Of all families, 22 carried *MLH1*, 12 *MSH2*, and 61 unknown for mutation status.  [Ascertainment of families was not described.] | Retrospective cohort study | Overall  O=55  SIR=13.38 (95% CI 9.4-19.0)  Mean age at diagnosis=54.27 years  *MLH1* carriers  O=9  SIR=14.77 (95% CI 6.2-35.0)  Mean age at diagnosis=51.33 years  *MSH2* carriers  O=2  SIR=2.02 (95% CI 0.3-12.7)  Mean age at diagnosis=54 years  Mutation negatives  O=44  SIR=18.03 (95% CI 12.2-26.7)  Mean age at diagnosis=55.55 years  [The statistical method for SIR calculation was not reported.] | Ascertainment of families was not described; and estimate of association may be upwardly biased. ^¶¶^ |
| Barrow et al. | 2009 | UK | 249 confirmed mutation carriers (105 *MLH1*, 133 *MSH2*, 11 *MSH6*) and 90 obligate^#^ carriers (39 *MLH1*, 46 *MSH6*, 5 *PMS2*)  Ascertained from families fulfilling the Amsterdam or Bethesda criteria who attended the Manchester  Regional Genetics Service. | Retrospective cohort study | O=25  E=not reported  SIR=not reported  Cumulative risk to age 70 years  *MLH1*: 18.2% (95% CI 11.9-24.5)  *MSH2*: 1.5% (95% CI 0-3.0)  Population: 7.5-8% | Estimate of association may be upwardly biased. ^¶¶^ |
| Engel et al. | 2012 | Germany  Netherlands | 2,118 confirmed (806 *MLH1*, 1004 *MSH2*, and 308 *MSH6*) (1107 women).  Ascertained from families fulfilling the Amsterdam or Bethesda criteria through the German HNPCC Consortium and the registry of the Netherlands Foundation for the Detection of Hereditary Tumors. | Retrospective cohort study | O=50  E=not reported  SIR=1.9 (95% CI 1.4-2.4)  Cumulative risk to age 70 years=14.4% (95% CI 9.5-19.3)  Median age at diagnosis=52 years (range 30-76) | Estimate of association may be upwardly biased. ^¶¶^ |
| Baglietto et al. | 2010 | USA  Canada  Australia  New Zealand  Netherlands  Scotland | 3,104 members from 113 families with *MSH6* mutation.  Ascertained from population cancer registries or family cancer clinics of the Colon Cancer Family Registry and other European sites. | Retrospective cohort study | O=25  HR= 0.6 (95% CI 0.2- 1.6) | Ascertainment was corrected by statistical methods conditioning the likelihood for each pedigree.  The estimates may reflect for *MSH6* mutation carriers only. |
| Dowty et al. | 2013 | USA  Canada  Australia  New Zealand | 17,576 members from families with *MLH1* (n=166) or *MSH2* (n=224) mutations.  Ascertained from population cancer registries or family cancer clinics of the Colon Cancer Family Registry. | Retrospective cohort study | *MLH1* carriers  O=53  HR=1.1 (95% CI 0.47-2.6)  Mean age at diagnosis = 55.4 (SD 13.8)  *MSH2* carriers  O=102  HR=1.5 (95% CI 0.71-3.3)  Mean age at diagnosis = 55.4 (SD 14.5) | Ascertainment was corrected by statistical methods conditioning the likelihood for each pedigree.  The estimates may reflect for *MLH1* or *MSH2* mutation carriers only. |
| Win et al. | 2012 | USA  Canada  Australia  New Zealand | 446 confirmed carriers (161 *MLH1*, 222 *MSH2*, 47 *MSH6* and 16 *PMS2*).  A median 5 years follow-up. Ascertained from population cancer registries or family cancer clinics of the Colon Cancer Family Registry. | Prospective cohort study | O=7  E=1.77  SIR=3.95 (95% CI 1.59-8.13)  Median age at diagnosis=56 years (range 42-62)  Cumulative risk: 1% (0.3-3%) at 5 years and 4% (2-11%) at 10 years | No ascertainment bias.  Breast cancer risk may be attributed by screening detection. |
| Win et al. | 2012 | USA  Canada  Australia  New Zealand | 764 confirmed carriers (316 *MLH1*, 357 *MSH2*, 49 *MSH6* and 42 *PMS2*) who had a previous diagnosis of colorectal cancer.  Ascertained from population cancer registries or family cancer clinics of the Colon Cancer Family Registry. Probands were not ascertained because they had had multiple cancers. | Retrospective cohort study | All carriers combined  O=20  E=11.34  SIR=1.76 (95% CI 1.07-2.59)  Median age at diagnosis=60 years (range 43-79)  Cumulative risk = 2% (95% CI 0.6-4%) at 10 years and 11% (95% CI 1-17%) at 20 years following colorectal cancer  *MLH1* carriers  O=5  E=5.08  SIR=0.99 (95% CI 0.22-1.98)  *MSH2* carriers  O=13  E=5.52  SIR=2.36 (95% CI 1.19-3.73)  *MSH6* carriers  O=2  E=0.41  SIR=4.90 (95% CI 0-13.03) | No ascertainment bias.  The estimates may reflect for mutation carriers who had a previous diagnosis of colorectal cancer only. |
| Win et al. | 2013 | USA  Canada  Australia  New Zealand | 127 confirmed carriers (30 *MLH1*, 72 *MSH2*, 22 *MSH6* and 3 *PMS2*) who had a previous diagnosis of endometrial cancer.  Ascertained from population cancer registries or family cancer clinics of the Colon Cancer Family Registry. Probands were not ascertained because they had had multiple cancers. | Retrospective cohort study | All carriers combined  O=12  E=4.79  SIR=2.51 (95% CI 1.17-4.14)  Median age at diagnosis=63 years (range 37-80)  Cumulative risk = 5% (95% CI 1-10%) at 10 years and 11% (95% CI 4-19%) at 20 years following endometrial cancer.  *MLH1* carriers  O=2  E=1.16  SIR=1.72 (95% CI 0-4.21)  *MSH2* carriers  O=7  E=2.92  SIR=2.39 (95% CI 0.82-4.47)  *MSH6* carriers  O=3  E=0.62  SIR=4.84 (95% CI 0-11.66) | No ascertainment bias.  The estimates may reflect for mutation carriers who had a previous diagnosis of endometrial cancer only. |
| Blokhuis et al. | 2008 | Brazil | 87 mutation-positive females vs 121 mutation-negative sisters of MLH1 c.C1528T mutation  Ascertained via colorectal cancer cases diagnosed age <50 from the Colorectal Surgery Unit at Groote Schuur Hospital and the Division of Human Genetics at the University of Cape Town, South Africa. | Case-control | O=7/87 (8%) in mutation-positive females vs 4/121 (3%) in mutation-negative sisters; p=0.21 | No ascertainment bias.  The estimates may reflect *MLH1* c.C1528T mutation carriers only. |

SIR=standardized incidence ratio, CI=confidence interval, MMR=mismatch repair, O=observed number of breast cancer, E=expected number of breast cancer, HNPCC=hereditary non-polyposis colorectal cancer, SD=standard deviation, OR=odds ratio.

^¶^ SIR was calculated dividing the observed number of death from breast cancer by the expected number of death .

*Putative carrier was defined as HNPCC family members affected by any cancer .

**Obligate carrier was not defined .

^┼^ Obligate carrier was not defined .

^┼┼^ Phenotypic carrier was defined as individual who was not confirmed mutation or obligate carrier but who had a type of cancer or combination of cancers at an age strongly suggestive of them being carrier .

^ Odds ratio was defined by (number of breast cancer in affected individuals/number of other cancers in affected individuals)/(number of breast cancer in general population/ number of other cancers in general population) .

^#^ Obligate carrier was defined because of position in the pedigree in relation to a confirmed MMR gene mutation carrier .

^##^ Putative carrier was not defined .

^^ Obligate carrier was defined under the assumption that the deleterious mutations observed in the members of a single kindred were identical by descent .

^¶¶^ Estimates of breast cancer risk are likely to be upwardly biased if any of the family members attended the clinics because of a family history of breast cancer (see text for details).
